# Clinical remission with biologic therapies in severe asthma: a matter of definition

**DOI:** 10.1183/13993003.00160-2024

**Published:** 2024-06-20

**Authors:** On behalf of the UK Severe Asthma Registry, P. Jane McDowell, Ron McDowell, John Busby, M. Chad Eastwood, Pujan H. Patel, David J. Jackson, Adel Mansur, Mitesh Patel, Hassan Burhan, Simon Doe, Rekha Chaudhuri, Robin Gore, James W. Dodd, Deepak Subramanian, Thomas Brown, Liam G. Heaney

**Affiliations:** 1 Wellcome Wolfson Centre for Experimental Medicine, School of Medicine, Dentistry and Biomedical Sciences, Queen's University, Belfast, UK; 2 Belfast Health and Social Care NHS Trust, Belfast, UK; 3 Centre for Public Health, School of Medicine, Dentistry and Biomedical Sciences, Queen's University, Belfast, UK; 4 Royal Brompton and Harefield Hospitals, London, UK; 5 Guys Severe Asthma Centre, Guy's Hospital, School of Immunology and Microbial Sciences, King's College London, London, UK; 6 University of Birmingham and Heartlands Hospital, Birmingham, UK; 7 Department of Respiratory Medicine, University Hospitals Plymouth NHS Trust, Derriford Hospital, Plymouth, UK; 8 Royal Liverpool University Hospital, Liverpool, UK; 9 The Newcastle upon Tyne Hospitals NHS Foundation Trust, Newcastle Upon Tyne, UK; 10 NHS Greater Glasgow and Clyde Health Board, Gartnavel Hospital, Glasgow, UK; 11 Addenbrooke's Hospital, Cambridge University Hospitals NHS Foundation Trust, Cambridge, UK; 12 Academic Respiratory Unit, University of Bristol, Bristol, UK; 13 University Hospitals of Derby and Burton NHS Foundation Trust, Derby, UK; 14 Portsmouth Hospitals NHS Trust, Portsmouth, UK

## Abstract

We read with interest the editorial “Clinical remission with biologic therapies in severe asthma: a matter of definition” [1]. We absolutely agree that the definition of clinical remission is of critical importance, and as with rheumatology, gastroenterology and dermatology, this is likely to be an iterative process. Early intervention with targeted treatments has been associated with an improvement in quality of life and decreased symptom burden in these non-respiratory inflammatory diseases, and certainly the onus to provide prospective evidence showing improved quality of life and disease outcomes with early intervention and sustained remission in severe asthma now lies with the severe asthma community.

## Shareable abstract

**There is currently no evidence to support the use of maintenance and reliever therapy (MART) in patients with severe asthma and persistently elevated T2 biomarkers despite adherence to high dose ICS treatment** https://bit.ly/42IOVbA

*To the Editor*:

We read with interest the editorial “Clinical remission with biologic therapies in severe asthma: a matter of definition” [1]. We absolutely agree that the definition of clinical remission is of critical importance, and as with rheumatology, gastroenterology and dermatology, this is likely to be an iterative process. Early intervention with targeted treatments has been associated with an improvement in quality of life and decreased symptom burden in these non-respiratory inflammatory diseases, and certainly the onus to provide prospective evidence showing improved quality of life and disease outcomes with early intervention and sustained remission in severe asthma now lies with the severe asthma community.

However, we were surprised by the authors’ assertion that the patients in the UK Severe Asthma Registry (UKSAR) are not “optimised” because they were not on maintenance and reliever therapy (MART), and therefore were not eligible for biologic therapy as per National Institute for Health and Care Excellence (NICE) criteria.

The editorial references a network meta-analysis [2] which included a subset analysis of 10 MART studies in “moderate–severe” asthma, and a meta-analysis of five papers which focuses on selected participants within MART studies with persistent asthma symptoms (Asthma Control Questionnaire score >1.5) [3]. The baseline features of the cohorts included in these meta-analyses and the UKSAR population are outlined in table 1 [4–16].

**TABLE 1:** Patient characteristics at study entry

Study	N	Age, years	FEV_1_, %	ACQ	Exacerbations in 1 year	Blood eosinophils, ×10^9^	*F*_ENO_, ppb	Adherence assessment	mOCS	LABA	ICS treatment: baseline ICS µg·day^−1^		
**McDowell [** **4** **]**	1111	52.0 (41–61)	66.9 (52–82)	3.2 (2–4)	5 (3–8)	0.70 (0.44–1.10)	43.0 (24–75)	MPR, *F*_ENO_ suppression, cortisol/prednisolone levels	58% (638)	93% (1013)	2000 (1600–2000)^+^		
**Rogliani [2] sub-analysis of 10 studies with moderate–severe asthma in a MART network analysis**											**Treatment group 1**	**Treatment group 2**	**Treatment group 3**
Pavord [5]	127	40	81	NR	NR	NR^#^	22.4	NR	0	82.5%	867 (200–1600)^+^	741 (200–1600)^+^	
Sovani [6]	71	40.3	85.2	2	1.09	NR	NR	MPR	0	NR	565±254	611±222	
Takeyama [7]	63	40	69.3	NR	≥1	NR^#^	NR	Self-report	0	50.8%	574±84	610±92	
Lin [8]	222	49.7	63.7	2.07	1.65	NR	NR	NR	0	53.5%	721 (400–1500)^+^	701 (400–1000)^+^	
Bousquet [9]	2309	39.5	70.6	NR	1.85	NR	NR	Self-report	0	55%	720 (200–2000)^+^	705 (250–1600)^+^	
Kuna [10]	3335	38	72.7	NR	≥1	NR	NR	Self-report	0	47%	744±230	750±262	
Rabe [11]	3382	38	72	1.9	≥1	NR	NR	Self-report	0	59%	751 (250–1600)^+^	757 (160–1600)^+^	758 (320–1600)^+^
Vogelmeier [12]	2143	45	73	1.9	≥1	NR	NR	NR	0	38%	881 (400–3000)^+^	888 (50–2000)^+^	
O'Byrne [13]	2760	35.6	73	NR	≥1	NR	NR	NR	0	28%	620 (100–1000)^+^	598 (200–1000)^+^	619 (200–1200)^+^
Scicchitano [14]	1890	43	70	NR	≥1	NR	NR	Self-report	0	35%	744 (250–2000)^+^	748 (400–2000)^+^	
**Beasley [3]: sub-analysis of study participants with high ACQ score within study groups**											**GINA 3**	**GINA 4**	
Bousquet [9]	1419	39.2±16	69.63±13.35	2.43±0.68	NR^¶^	NR	NR	Self-report	0	NR^¶^	52.4% (744)	47.6% (675)	
Kuna [10]	2160	38.4±17	72.37±13.72	2.51±0.70	≥1	NR	NR	Self-report	0	NR^¶^	53.1% (1146)	46.9% (1014)	
Patel [15]	154	40.1±14	78.03±19.20	2.59±0.85	NR^¶^	NR	NR	Electronic monitoring	0	NR^¶^	39.0% (60)	61.0% (94)	
Atienza [16]	466	46.1±15	70.65±14.63	2.40±0.68	NR^¶^	NR	NR	NR	0	NR^¶^	100% (466)	0	
Rabe [11]	664	41.3±16.2	72.07±12.83	2.37±0.61	NR^¶^	NR	NR	Self-report	0	NR^¶^	100% (664)	0	
**Total N for GINA step 3 and 4**											63.3% (3080)	36.7% (1783)	

Data are presented as mean±sd or median (interquartile range), as appropriate, except where indicated with “+”; these data are presented as mean (minimum and maximum values). FEV_1_: forced expiratory volume in 1 s; ACQ: Asthma Control Questionnaire; *F*_ENO_: fractional exhaled nitric oxide; mOCS: maintenance oral corticosteroids; LABA: long-acting β-agonist; ICS: inhaled corticosteroid; MPR: medicines possession ratio; MART: maintenance and reliever therapy; GINA: Global Initiative for Asthma; NR: none recorded. ^#^: sputum eosinophils but not blood eosinophils recorded; ^¶^: characteristics for the whole study cohort may be presented in the original paper, but data for the subset of patients included in the high ACQ analysis are not published.

The UKSAR cohort had severe asthma with high dose inhaled corticosteroid (ICS)/long-acting β-agonist (LABA) (plus additional controllers), high symptom burden, high exacerbation count and persistently elevated T2 biomarkers despite objective evidence of adherence to background treatment (high medicines possession ratio, cortisol/prednisolone levels and digital inhaler monitoring as recommended by the European Respiratory Society/American Thoracic Society guidelines [17]), and notably 58% were receiving maintenance prednisolone. These patients meet the criteria for the definition of severe asthma (box 1).

**BOX 1:** Severe asthma definitions

**European Respiratory Society/American Thoracic Society [17]:** 1) High dose ICS (>1000 μg·day^−1^)^#^, plus LABA or LTRA/theophylline, or mOCS ≥50% of the time 2) Uncontrolled asthma (ACQ >1.5 AND ≥2 OCS-requiring exacerbations or ≥1 exacerbation requiring hospitalisation AND airflow limitation) OR controlled asthma, worsening on OCS weaning 3) Comorbidity assessment and management to differentiate from difficult to treat asthma, including assessment of nonadherence and inhaler technique
**Global Initiative for Asthma (GINA) [18]:** 1) GINA step 4–5 (step 4: medium–high dose ICS/LABA combination (>500 μg·day^−1^ ICS^#^); step 5: consideration of high dose ICS/LABA (>1000 μg ICS) combination plus additional controllers/biologics) 2) Poor symptom control 3) Good adherence and inhaler technique

^#^: in ≥12-year-olds. ICS: inhaled corticosteroid; LABA: long-acting β-agonist; LTRA: leukotriene receptor antagonist; (m)OCS: (maintenance) oral corticosteroid; ACQ: Asthma Control Questionnaire.

The moderate–severe asthma cohorts represented in the meta-analysis [2] and the sub-cohort of patients with a high symptom burden in the Beasley *et al.* [3] analysis bear little resemblance to the patients in the UKSAR paper: 1) the mean ICS dose in the network sub-analysis was ∼700 μg·day^−1^ with only 50% receiving additional LABA for asthma control [2], and 63% of patients in the 2022 meta-analysis were GINA step 3 (*i.e.* receiving only low dose ICS/LABA) [3]; 2) only two studies used objective measures to assess patient adherence; 3) the baseline exacerbation rate into many of these studies was one; and importantly 4) there was no measurement of T2 biomarkers to assess if these were cohorts that had unsuppressed T2 inflammation despite baseline therapy.

Thus, the patient population in UKSAR are minimally represented in the MART studies cited by Beasley *et al.* [1]*.* We would suggest there is no evidence that a MART approach will have any beneficial impact on the disease burden in UKSAR, and in fact there is robust evidence that patients with persistently uncontrolled T2 inflammation on medium dose ICS have increased exacerbation rates and a lower forced expiratory volume in 1 s when compared to high dose ICS [19, 20].

In summary, while we acknowledge the current enthusiasm for MART therapy, the suggestion that MART would be advantageous in this specific clinical population has no evidence base. We would caution against generalisations from studies in broader populations of moderate–severe asthma to this well-defined severe asthma cohort that have persistently elevated T2 biomarkers despite guideline-directed high dose ICS and LABA therapy. We strongly refute the suggestion that these patients were not “optimised”, indeed this is mandated by the NICE access criteria which requires adherence with “…high dose ICS and additional controllers…” which was the case in all UKSAR patients prior to the use of biologic therapy.

## Shareable PDF

10.1183/13993003.00160-2024.Shareable1 This one-page PDF can be shared freely online.
Shareable PDF ERJ-00160-2024.Shareable
