# Supplementary material for: Clinical remission with biologic therapies in severe asthma: a matter of definition
Source: Eur Respir J. 2024 Jun 20;63(6):2400160. doi: 10.1183/13993003.00160-2024 (PMC11187314; doi:10.1183/13993003.00160-2024)
Supplement: Supplementary file 1 [file ERJ-00160-2024.Shareable.pdf]

EUROPEAN RESPIRATORY JOURNAL
CORRESPONDENCE
P.J. MCDOWELL ET AL.

SHAREABLE PDF

Clinical remission with biologic therapies in severe asthma: a matter of definition

On behalf of the UK Severe Asthma Registry

On behalf of the UK Severe Asthma Registry, P. Jane McDowell [1,2], Ron McDowell [3], John Busby [3], M. Chad Eastwood [1,2], Pujan H. Patel [4], David J. Jackson [5], Adel Mansur [6], Mitesh Patel [7], Hassan Burhan [8], Simon Doe [9], Rekha Chaudhuri [10], Robin Gore [11], James W. Dodd [12], Deepak Subramanian [13], Thomas Brown [14] and Liam G. Heaney [1,2]

[1]Wellcome Wolfson Centre for Experimental Medicine, School of Medicine, Dentistry and Biomedical Sciences, Queen's University, Belfast, UK. [2]Belfast Health and Social Care NHS Trust, Belfast, UK. [3]Centre for Public Health, School of Medicine, Dentistry and Biomedical Sciences, Queen's University, Belfast, UK. [4]Royal Brompton and Harefield Hospitals, London, UK. [5]Guys Severe Asthma Centre, Guy's Hospital, School of Immunology and Microbial Sciences, King's College London, London, UK. [6]University of Birmingham and Heartlands Hospital, Birmingham, UK. [7]Department of Respiratory Medicine, University Hospitals Plymouth NHS Trust, Derriford Hospital, Plymouth, UK. [8]Royal Liverpool University Hospital, Liverpool, UK. [9]The Newcastle upon Tyne Hospitals NHS Foundation Trust, Newcastle Upon Tyne, UK. [10]NHS Greater Glasgow and Clyde Health Board, Gartnavel Hospital, Glasgow, UK. [11]Addenbrooke's Hospital, Cambridge University Hospitals NHS Foundation Trust, Cambridge, UK. [12]Academic Respiratory Unit, University of Bristol, Bristol, UK. [13]University Hospitals of Derby and Burton NHS Foundation Trust, Derby, UK. [14]Portsmouth Hospitals NHS Trust, Portsmouth, UK.

Corresponding author: Liam G. Heaney (l.heaney@qub.ac.uk)

Check for updates

Shareable abstract (@ERSpublications)
There is currently no evidence to support the use of maintenance and reliever therapy (MART) in patients with severe asthma and persistently elevated T2 biomarkers despite adherence to high dose ICS treatment https://bit.ly/42IOVbA

Cite this article as: . Clinical remission with biologic therapies in severe asthma: a matter of definition. *Eur Respir J* 2024; 63: 2400160 [DOI: 10.1183/13993003.00160-2024].

This extracted version can be shared freely online.

Copyright ©The authors 2024.

This version is distributed under the terms of the Creative Commons Attribution Licence 4.0.

Received: 22 Jan 2024
Accepted: 11 Feb 2024

To the Editor:

We read with interest the editorial "Clinical remission with biologic therapies in severe asthma: a matter of definition" [1]. We absolutely agree that the definition of clinical remission is of critical importance, and as with rheumatology, gastroenterology and dermatology, this is likely to be an iterative process. Early intervention with targeted treatments has been associated with an improvement in quality of life and decreased symptom burden in these non-respiratory inflammatory diseases, and certainly the onus to provide prospective evidence showing improved quality of life and disease outcomes with early intervention and sustained remission in severe asthma now lies with the severe asthma community.
